# Efficacy of a novel chewable tablet (Credelio Quattro™) containing lotilaner, moxidectin, praziquantel, and pyrantel for the treatment and control of hookworm infections in dogs

**DOI:** 10.1186/s13071-025-06757-4

**Published:** 2025-04-02

**Authors:** Samuel Charles, Katrin Deuster, Xinshuo Wang, Scott Wiseman, Craig R. Reinemeyer, Luther van der Mescht, Abdelmoneim Mansour, Imad Bouzaidi Cheikhi, Lisa Young

**Affiliations:** 1 Elanco Animal Health, 2500 Innovation Way, Greenfield, IN 46140 USA; 2 Elanco Animal Health GmbH, Alfred-Nobel-Str. 50, 40789 Monheim, Germany; 3 Elanco Animal Health, Form 2, Bartley Way, Bartley Wood Business Park, Hook, RG27 9XA UK; 4 East Tennessee Clinical Research Inc, Rockwood, TN USA; 5 Clinvet, Uitzich Road, Bainsvlei, Bloemfontein, 9338 South Africa; 6 TRS Labs, Inc., Athens, GA 30607 USA; 7 Clinvet, Douar Dbabej, Beni Yekhlef, 28815 Mohammedia, Morocco; 8 Department of Zoology and Entomology, University of the Free State, Bloemfontein, South Africa

**Keywords:** Moxidectin, Pyrantel, Efficacy, Hookworm, *Ancylostoma caninum*, *Uncinaria stenocephala*

## Abstract

**Background:**

Hookworms, specifically *Ancylostoma caninum* and *Uncinaria stenocephala*, have a clinical impact on the health of dogs, with *A. caninum* posing a zoonotic risk worldwide. The studies presented here were conducted to evaluate the efficacy of a novel, oral chewable tablet (Credelio Quattro) containing lotilaner, moxidectin, praziquantel, and pyrantel (as pamoate salt) against fourth-stage larvae (L_4_), immature adult, and adult *A. caninum*, as well as adult *U. stenocephala*, infections in dogs.

**Methods:**

Nine negatively controlled, masked, randomized laboratory studies evaluated the efficacy and non-interference of the drugs against various stages of *A. caninum* and *U. stenocephala*. In addition to one pilot study conducted against L_4_ *A*. *caninum* and one study that assessed efficacy in dogs with naturally acquired *U. stenocephala*, two experimental studies were conducted against each of the target hookworm species and stages. A total of 16–31 dogs comprised each study. With the exception of the study in dogs with naturally acquired *U. stenocephala*, dogs were experimentally infected with the target parasite and dosed on Day 0 or 4 with placebo tablets, Credelio Quattro tablets (or components of Credelio Quattro formulation for the pilot study), or individual actives, moxidectin or pyrantel, in the specific studies designed to assess interference. Efficacy was evaluated by comparing the number of worms recovered at necropsy 5–10 days post-treatment between the treated and control groups.

**Results:**

All dogs tolerated Credelio Quattro well. Efficacy of Credelio Quattro was ≥ 99.0% against L_4_ *A. caninum*, ≥ 99.8% against immature adult *A. caninum*, ≥ 99.9% against adult *A. caninum*, and ≥ 99.6% against adult *U. stenocephala.* Additionally, treatment with Credelio Quattro provided a ≥ 99.9% reduction in fecal egg counts 10 days post-treatment.

**Conclusions:**

Credelio Quattro, a novel oral chewable tablet administered at the minimum effective dosages of 20 mg/kg lotilaner, 0.02 mg/kg moxidectin, 5.0 mg/kg praziquantel, and 5.0 mg/kg pyrantel (as pamoate salt), was safe and effective for the treatment and control of L_4_, immature adult, and adult stages of *A. caninum* and adult *U. stenocephala* in dogs.

**Graphical Abstract:**

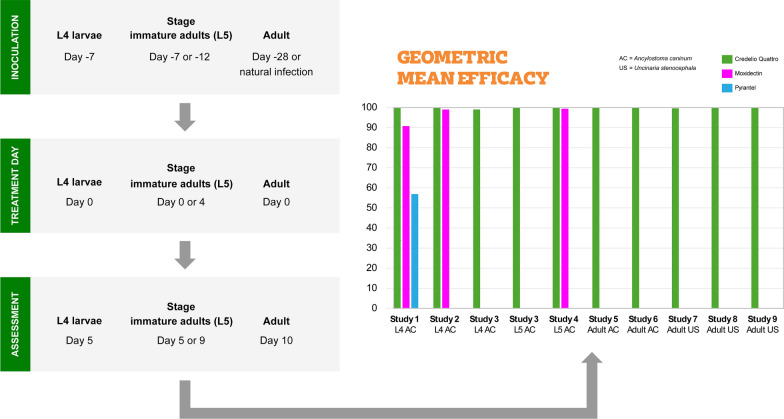

## Background

Hookworms are a significant global concern for pet health, with species such as *Ancylostoma caninum, Ancylostoma braziliense, Ancylostoma ceylanicum*, and *Uncinaria stenocephala* causing widespread infections in dogs and cats [1]. Among these, *A. caninum* and *U. stenocephala* are of particular concern due to their high prevalence and substantial impact on the health of dogs. While *Ancylostoma* spp. are predominantly found in warmer climates, *U. stenocephala*, also known as the northern hookworm, is more common in colder and temperate regions [2]. A recent study conducted in the USA that analyzed more than 39 million fecal samples from 2012 to 2018 showed a consistent increase in hookworm prevalence from 2015, demonstrating a total upsurge of 47% [3, 4]. In Europe, prevalence rates of hookworm infections in dogs vary widely, ranging from 1.2% to 34% [5].

Hookworm infections in the small intestine of dogs can present a range of clinical signs, depending on the infection intensity, hookworm species, and age of the dog. Hookworms, particularly *A. caninum*, are voracious blood feeders and can cause severe health conditions such as anemia, weight loss, and substantial gastrointestinal disturbances, including blood loss through the intestinal tract and fatalities in young dogs [6, 7]. Clinical signs may include the presence of occult blood in feces, bloody diarrhea (melena), and in severe cases, puppies may even succumb to extensive blood loss and malnutrition [7]. From a veterinary perspective, these symptoms stem from the hookworms’ ability to cause anemia and hypoproteinemia in their canine hosts. Notably, *A. caninum* causes significantly higher blood loss (1–2 mL per worm per day) [8], compared with *A. ceylanicum* (0.033 mL per worm per day), *A. braziliense* (0.002 mL per worm per day), and *U. stenocephala* (0.0003 mL per worm per day) [2, 9, 10]. In hookworm species with a lung migration phase such as *A. caninum*, symptoms such as coughing, nasal discharge, fever, and further signs related to pneumonia can also occur. Hookworms not only affect dog health and welfare, but also have zoonotic potential, making them a significant public health concern. In humans, contact with infective larvae from contaminated soil can lead to a self-limiting local dermatitis known as cutaneous larva migrans (CLM), caused by migrating larvae burrowing through the upper dermis [11, 12]. Furthermore, the pre-adult stage of *A. caninum* is widely acknowledged as a causative factor of eosinophilic enteritis in humans [13].

*Ancylostoma caninum* has a lifecycle that commences with adult worms inhabiting the small intestine [14, 15]. The female hookworm releases and expels thousands (2000–17,000) of eggs per day into the environment through the host’s feces. These eggs hatch within 1–2 days into first-stage larvae (L_1_) in the soil. Within 5–10 days, these larvae develop into infective third-stage larvae (L_3_). The L_3_ can infect dogs either by being ingested directly from the environment or from paratenic hosts (e.g., rodents, cockroaches) or by penetrating the skin [16]. Ingested larvae develop into adult worms directly in the intestine, while skin-penetrating larvae migrate through the tissues before reaching the intestine and maturing into adults. Additionally, in puppies, lactogenic transmission of *A. caninum* is a major source of infection. Hookworm larvae can undergo somatic migration and persist as L_3_ in an arrested state in the tissues for years, reactivating during pregnancy and migrating to the mammary glands [7, 17]. Once matured, adult hookworms feed on mucous membranes with the different intensities of blood loss described above and produce eggs, completing the lifecycle within 2–3 weeks. (Lifecycle image: pet.elanco.com/us/en/interceptorplus/hookworm-lifecycle.)

Current recommendations for control and prevention of hookworms as provided by the Companion Animal Parasite Council (CAPC) in the USA, and the Tropical Council for Companion Animal Parasites (TroCCAP) in the tropics include treating puppies with anthelmintics at 2-week intervals, from 2 weeks of age through 8 weeks of age, and then with a monthly preventive thereafter. If initial treatment is delayed, puppies should be dewormed 2 weeks following the first preventive dose and then continue regular monthly preventive through a year-round broad spectrum deworming program according to CAPC [18]. In the first year of life, CAPC recommends dogs receive fecal examinations at least 2–4 times and then 1–2 times annually thereafter [18], and TroCCAP recommends dogs are tested at least every 3 months [19]. The European Scientific Counsel Companion Animal Parasites (ESCCAP) recommends testing and deworming protocols on the basis of individual risk assessments, with up to monthly treatments for dogs in the highest risk groups [20, 21].

The second-generation macrocyclic lactone moxidectin, belonging to the milbemycin family, is a proven parasiticide for dogs available in different formulations and dosages, notably as a topical solution (Advantage Multi™/Advocate™, Elanco Animal Health) and an oral tablet (Simparica Trio^®^, Zoetis). Pyrantel in the form of the pamoate salt (or embonate salt) is well established at a minimum effective dosage of 5.0 mg/kg in veterinary medicine and is authorized in oral combination formulations as treatment for nematodes in dogs (e.g., Simparica Trio; NexGard^®^ PLUS, Boehringer Ingelheim Animal Health USA, Inc, Duluth, GA, USA).

The objective of the studies described here was to evaluate the efficacy of a novel, oral chewable tablet containing lotilaner, moxidectin, praziquantel, and pyrantel (Credelio Quattro, Elanco Animal Health, Greenfield, IN, USA), against induced L_4_, immature adult, and adult *A. caninum*, as well as induced and natural infections of adult *U. stenocephala* in dogs.

### Methods

In total, nine masked, randomized laboratory studies were conducted. Studies were conducted according to VICH GL9 Good Clinical Practice or, in the case of the pilot L_4_ study, Good Research Practice, VICH GL19 Effectiveness of Anthelmintics: Specific Recommendations for Canine [22] and VICH GL7 Effectiveness of Anthelmintics: General Recommendations, and World Association for the Advancement of Veterinary Parasitology (WAAVP) second edition: Guidelines for evaluating the efficacy of anthelmintics for dogs and cats [23].

### Animals

Purpose-bred laboratory Beagles or mixed breed dogs in good health after a physical examination at enrollment were selected. Dogs ranged in age from 2 to 13 months at the time of hookworm inoculation and body weights ranged from 2.75 to 22.4 kg at the time of treatment administration. With the exception of the natural *U. stenocephala* infection study, dogs were pair-housed until allocation to treatment groups and individually housed starting on Day −1. Housing conformed to appropriate animal welfare standards. Dogs were fed a commercial diet suitable for their age and were provided potable water ad libitum. General health observations were conducted at least once daily for the duration of each study.

### Design

A total of nine studies were conducted to evaluate the efficacy of a combination tablet of lotilaner, moxidectin, praziquantel, and pyrantel (LMPP) against different stages (L_4_, immature adult, adult) of *A. caninum* and adult *U. stenocephala* from the USA, European Union (EU), and Morocco (MAR). Studies 1, 2, and 3 evaluated the efficacy against larvae (L_4_); Studies 3 and 4 assessed immature adults; and Studies 5 and 6 examined adult *A. caninum* efficacy. Studies 7, 8, and 9 assessed efficacy against adult *U. stenocephala*. In addition to efficacy, Study 1 included an assessment of which active component in the LMPP combination (moxidectin or pyrantel) was responsible for efficacy against L_4_ *A. caninum* and included a group treated with moxidectin alone and pyrantel alone. Studies 2 and 4 included an investigation of whether the combination of individual active components interfered with the efficacy of moxidectin against L_4_ and immature adult *A. caninum* and included groups treated with moxidectin alone.

Except for Study 9, in which dogs were naturally infected with *U. stenocephala*, all studies were performed with experimentally induced infections. Study 9 also employed cohorts, defined by the time of enrollment of an *U. stenocephala*-positive dog in the study.

For Study 1 (a pilot study), the combination LMPP product was administered in gelatin capsules. Studies 2–9 used Credelio Quattro in the final commercial chewable tablet formulation.

### Experimentally induced and natural hookworm infections

For experimentally induced studies, efficacy against each stage of *A. caninum* and *U. stenocephala* was evaluated against isolates collected in the EU and USA. In the US studies, three isolates of *A. caninum* were obtained from naturally infected dogs in Tennessee (2019–2021) and *U. stenocephala* was sourced from a naturally infected dog in Kentucky (2016). The *A. caninum* used in the EU studies originated from Romania (2018) and the *U. stenocephala* was isolated from naturally infected dogs in southern Italy (2016). The isolates were maintained by inoculation of donor dogs as required. Inoculum size and timing between inoculation of dogs and dosing was set on the basis of the known lifecycle of the parasite to develop into the target stage as per guidelines [22] and is summarized in Table 1.

**Table 1:** Study designs to assess the efficacy of Credelio Quattro against hookworm infections in dogs

Study	Parasite	Isolate origin	Stage at time of treatment	Day of inoculation	Day of treatment	Day of worm recovery	Group	No. of animals
1	*A. caninum*	USA	L_4_	− 7	0	5	Control	6
							Combination pilot formulation	6
							Moxidectin	6
							Pyrantel	6
2	*A. caninum*	EU	L_4_	−7	0	5	Control	8
							Credelio Quattro	8
							Moxidectin	8
3	*A. caninum*	USA	L_4_	−7	0	5	Control	8
							Credelio Quattro	8
			Immature adult	−7	4	9	Control	8
							Credelio Quattro	8
4	*A. caninum*	EU	Immature adult	−12	0	5	Control	7
							Credelio Quattro	8
							Moxidectin	7
5	*A. caninum*	USA	Adult	−28	0	10	Control	10
							Credelio Quattro	10
6	*A. caninum*	EU	Adult	−28	0	10	Control	8
							Credelio Quattro	8
7	*U. stenocephala*	USA	Adult	−28	0	10	Control	10
							Credelio Quattro	10
8	*U. stenocephala*	EU	Adult	−28	0	10	Control	10
							Credelio Quattro	10
9	*U. stenocephala*	MAR	Adult	Natural infection	0	10	Control	13
							Credelio Quattro	18

In Studies 1, 2, and 3, which assessed the efficacy against L_4_ stages of *A. caninum*, dogs were orally inoculated with 300 *A. caninum* L_3_ 7 days before treatment. In Studies 3 and 4, which evaluated the efficacy against immature adult of *A. caninum*, dogs were orally inoculated with 300 *A. caninum* L_3_ 12 days before treatment. In Studies 5 and 6, which examined efficacy against adult *A. caninum*, dogs were orally inoculated with 300 *A. caninum* L_3_ 28 days before treatment. In Studies 7 and 8, which assessed efficacy against adult *U. stenocephala,* dogs were inoculated orally with 1000 *U. stenocephala* L_3_ 28 days before treatment. Food was withheld overnight pre-inoculation in all experimentally induced studies. Study 9 assessed the efficacy of *U. stenocephala* in naturally infected dogs that were positive by fecal egg count (FEC).

### Randomization and treatment

With the exception of Study 9, dogs were allocated randomly to treatment and housing according to a randomization and allocation plan, in a completely randomized design. Study 9 used cohorts; each cohort was made up of complete blocks of two dogs; dogs were allocated to treatment and housing in a randomized block design by cohort.

Unequal treatment assignment across gender was permitted. On Day –1 or Day –3, all dogs meeting the inclusion/exclusion criteria were assigned to treatment groups. For the adult *A. caninum* and adult *U. stenocephala* studies, dogs had to have a positive FEC (i.e., > 0 EPG) , assessed at least once between Day −4 and Day −1 to continue in the study.

On treatment day, dogs were dosed orally with either placebo or investigational veterinary product according to each study design (Table 1). Dogs received the appropriate combination of tablets to provide as close to the minimum effective dosages of 20 mg/kg lotilaner, 0.02 mg/kg moxidectin, 5.0 mg/kg praziquantel, and 5.0 mg/kg pyrantel on the basis of pre-treatment body weight. Placebo and active tablets or capsules were similar in appearance to maintain masking. Dosing was performed in a fed state.

### Fecal egg counts

In studies evaluating Credelio Quattro against adult stages of hookworm, pre-treatment quantitative FECs were conducted prior to allocation to treatment group. In Studies 5 and 6, FECs were performed at least twice during Days −4 to −2 to ensure the presence of a patent *A. caninum* infection in all dogs to be allocated to a treatment group. Similarly, in Studies 7 and 8, FECs were conducted prior to inclusion into the study to ensure presence of a patent *U. stenocephala* infection in all dogs. In Study 9, with dogs naturally infected with *U. stenocephala*, FECs were performed at least three times during Days −6 to −1 pre-treatment. Quantitative FECs were performed on Day 10 in Studies 5–9 to evaluate the percent reduction in FECs after administration of the novel combination tablet. Pre- and post-treatment FECs were performed using the McMaster method for Studies 5 and 9, or the Modified Wisconsin method for Studies 4, 6, 7, and 8.

### Worm recovery

Following humane euthanasia, necropsies were conducted on scheduled days as presented in Table 1. During necropsy, the abdominal cavity was opened and assessed for any abnormalities. The digestive tract was then carefully isolated and processed as per standard parasitological procedures. The entire gastrointestinal (GI) tract, from the stomach to the rectum, was extracted and placed into a dedicated container. The GI tract was split longitudinally, and the mucosal surface was scraped twice before being rinsed with tap water. The contents, scrapings, and rinse of the GI tract were washed over a sieve and then rinsed thoroughly with tap water. A #60 Standard Testing Sieve (250 µm openings) or a #100 Standard Testing Sieve (150 µm openings) was used for recovery of *A. caninum* and *U. stenocephala* respectively. For studies evaluating larval stages of *A. caninum* and the natural infections of *U. stenocephala*, an additional incubation step was completed in the recovery of worms. The isolated small intestine was incubated in a 0.9% NaCl solution at a temperature of > 90 °F for 2–4 h to facilitate separation of immature stages of the parasite from the mucosal tissues. After incubation, the small intestines were processed, washed, and sieved as described above. The contents of the container(s) were examined using additional light and magnification as required. All collected nematodes were identified, sexed, counted, and preserved in 10% buffered formalin. If worm fragments were present, the final count was based on the greater number of either heads or tails.

### Statistical analysis

The experimental unit for all studies reported here was the individual dog. A minimum of five worms needed to be present in each of at least six control dogs to show an adequate infection in the control group. Efficacy against *A. caninum* (L_4_, immature adult, adult) and adult *U. stenocephala* was the endpoint. Efficacy was determined post-treatment by comparison of the total *A. caninum* worm count (Studies 1–6), total *U. stenocephala* worm count (Studies 7 and 9), or total adult *U. stenocephala* worm count (Study 8) in the treated group versus the control group. A logarithmic transformation (ln[count + 1]) was applied to the post-treatment *A. caninum* and *U. stenocephala* worm counts for each individual animal to address the skewed nature of the data and stabilize the variance. The transformed counts were analyzed using an analysis of variance (ANOVA) model with a fixed effect for treatment. In Study 9, cohort was also accounted for as a random effect. Each treated group was compared with the control group in a separate statistical model (SAS® version 9.4), except for Study 1, in which all treatment groups were compared with control in a single model with the Dunnett adjustment.

Efficacy was defined as ≥ 90% reduction in the geometric mean (GM) counts of *A. caninum* (L_4_, immature adult, adult) and adult *U. stenocephala* in the treated group as compared with the negative control group. In addition, a statistically significant difference between the treatment group and the negative control group (*P* < 0.05, two-sided) was required. GM was estimated by back-transforming model least squares (LS) means. Adequacy of infection was defined as ≥ 5 worms in at least six control dogs according to VICH GL19 Effectiveness of Anthelmintics: Specific Recommendations for Canine [22].

In the studies that evaluated efficacy against adult hookworms (Studies 5, 6, 7, 8, and 9), the GM eggs per gram (EPG) pre-treatment and GM EPG 10 days post-treatment were calculated and the percent FEC reduction was reported for each treatment group.

## Results

All dogs tolerated Credelio Quattro well. A small number of animals in all groups, including controls, experienced non-serious, transient gastrointestinal signs. Table 2 summarizes efficacy results against L_4_, immature adult and adult stages of *A. caninum*, as well as adult *U. stenocephala*. Table 3 summarizes the percent reduction in FEC.

**Table 2:** Efficacy of a single oral dose of Credelio Quattro, pilot LMPP formulation, moxidectin alone, or pyrantel alone against hookworm infections in dogs

Study	Parasite	Isolate origin	Stage at time of treatment	Group	Geometric mean worm counts and efficacy		
					Worm count	% Efficacy	Statistical comparison with control group
1	*A. caninum*	USA	L_4_	Control	69.6	–	–
				Combination pilot formulation	0.1	99.8	*P* < 0.0001 (t_20_ = 14.45)
				Moxidectin	6.4	90.8	*P* < 0.0001 (t_20_ = 7.87)
				Pyrantel	30.0	56.9	*P* = 0.0249 (t_20_ = 2.87)
2	*A. caninum*	EU	L_4_	Control	168.8	–	–
				Credelio Quattro	0.4	99.8	*P* < 0.0001 (t_14_ = 19.72)
				Moxidectin	1.7	99.0	*P* < 0.0001 (t_14_ = 15.14)
3	*A. caninum*	USA	L_4_	Control	116.8	–	–
				Credelio Quattro	1.1	99.0	*P* < 0.0001 (t_14_ = 9.72)
			Immature adult	Control	125.3	–	–
				Credelio Quattro	0.3	99.8	*P* < 0.0001 (t_14_ = 15.92)
4	*A. caninum*	EU	Immature adult	Control	256.4	–	–
				Credelio Quattro	0.0	100	*P* < 0.0001 (t_13_ = 108.85)
				Moxidectin	1.5	99.4	*P* < 0.0001(t_12_ = 11.22)
5	*A. caninum*	USA	Adult	Control	227.3	–	–
				Credelio Quattro	0.0	100	*P* < 0.0001 (t_18_ = 182.28)
6	*A. caninum*	EU	Adult	Control	83.8	–	–
				Credelio Quattro	0.1	99.9	*P* < 0.0001 (t_14_ = 21.63)
7	*U. stenocephala*	USA	Adult	Control	478.5	–	–
				Credelio Quattro	1.8	99.6	*P* < 0.0001 (t_18_ = 21.10)
8	*U. stenocephala*	EU	Adult	Control	53.5	–	–
				Credelio Quattro	0.0	100	*P* < 0.0001 (t_18_ = 52.23)
9	*U. stenocephala*	MAR	Adult	Control	7.29	–	–
				Credelio Quattro	0.0	100	*P* < 0.0001 (t_24_ = 5.33)

**Table 3:** Percent reduction in *A. caninum* and *U. stenocephala* fecal egg counts 10 days post-treatment

Study	Parasite	Isolate origin	Stage at time of treatment	Group	Geometric mean EPG*		
					Pre-treatment	Post-treatment	% Reduction
5	*A. caninum*	USA	Adult	Control	5355.48	3926.55	–
				Credelio Quattro	4945.35	0.00	100
6	*A. caninum*	USA	Adult	Control	145.01	77.64	–
				Credelio Quattro	182.18	0.00	100
7	*U. stenocephala*	USA	Adult	Control	748.28	478.01	–
				Credelio Quattro	783.29	1.12	99.9
8	*U. stenocephala*	EU	Adult	Control	32.82	52.61	–
				Credelio Quattro	53.42	0.00	100
9	*U. stenocephala*	MAR	Adult	Control	211.06	111.42	–
				Credelio Quattro	255.37	0.00	100

^*^EPG, eggs per gram

### L_4_*Ancylostoma caninum*

In Studies 1, 2, and 3, all dogs in each control group had > 5 worms, confirming adequate infections to determine efficacy against L_4_ *A. caninum*. The GM *A. caninum* worm counts in the control groups were 69.6 (42–86), 168.8 (110–233), and 116.8 (40–214), respectively. In Study 1, the LMPP capsule-treated group had a GM worm count of 0.1, demonstrating an efficacy of 99.8%; in the group administered moxidectin alone, the GM worm count was 6.4, demonstrating an efficacy of 90.8%, and in the group administered pyrantel alone, the GM worm count was 30.0, resulting in 56.9% efficacy against L_4_ *A. caninum*. The worm counts in each of the treated groups were significantly different from the control group (*P* ≤ 0.0249). In Study 2, the Credelio Quattro- and moxidectin-treated groups had a GM worm count of 0.4 and 1.7, demonstrating 99.8% and 99.0% efficacy, respectively, with each group showing a statistically significant difference (*P* < 0.0001) in comparison with the control group. No significant difference was observed between the Credelio Quattro- and moxidectin alone-treated group, demonstrating non-interference of lotilaner, praziquantel, and pyrantel on the activity of moxidectin against L_4_ *A. caninum*. In Study 3, the Credelio Quattro-treated group had a GM worm count of 1.1, demonstrating 99.0% efficacy against L_4_ *A. caninum* with a statistically significant difference (*P* < 0.0001) when compared with the control group.

### Immature adult *Ancylostoma caninum*

In Studies 3 and 4, all dogs in each control group had an adequate infection (> 5 worms), with GM *A. caninum* worm counts of 125.3 (26–242) and 256.4 (210–312), respectively. In Study 3, the GM worm counts for Credelio Quattro-treated group were 0.3 demonstrating 99.8% efficacy against immature adult *A. caninum* and statistical significance as compared with the control group (*P* < 0.0001). In Study 4, the GM worm counts in the control group (256.4) were significantly different (*P* < 0.0001) when compared with zero worms in the Credelio Quattro group and 1.5 worms recovered in the moxidectin alone-treated group, demonstrating 100% and 99.4% efficacy, respectively, and confirmed non-interference of lotilaner, praziquantel, and pyrantel on the activity of moxidectin against immature adult *A. caninum*.

### Adult *Ancylostoma caninum*

In Studies 5 and 6, all dogs in each control group had > 5 worms, confirming adequate infections to determine efficacy against adult *A. caninum*. In Study 5, the GM worm counts for the control group and Credelio Quattro-treated group were 227.3 (202–270) and 0.0, and in Study 6, the GM worms counts were 83.8 (32–190) and 0.1 (0–1), respectively, demonstrating 100% and 99.9% efficacy of Credelio Quattro against adult *A. caninum* with a statistically significant difference (*P* < 0.0001) between the treated group and the control group in each study.

In Study 5, GM pre-treatment FECs were 5355.48 and 4945.35 in the control and Credelio Quattro groups, respectively; 10 days post-treatment, FECs in the control and Credelio Quattro groups were 3926.55 and 0.00, respectively, indicating a 100% reduction in FEC with Credelio Quattro. Similarly, in Study 6, the GM pre-treatment FECs were 145.01 and 182.18 in the control and Credelio Quattro groups, respectively, and 77.64 and 0.00, 10 days post-treatment, resulting in a 100% reduction in FEC post-treatment with Credelio Quattro.

### Adult *Uncinaria stenocephala*

In Studies 7 and 8, all 10 dogs in each control group had > 5 worms, confirming adequate infections to determine efficacy against adult *U. stenocephala*. In Study 7, the GM worm counts of the control group and the Credelio Quattro group were 478.5 (289–578) and 1.8 (0–8), respectively, and in Study 8, the GM worm counts were 53.5 (34–81) and 0.0, respectively, demonstrating 99.6% and 100% efficacy of Credelio Quattro against adult *U. stenocephala* with a statistically significant difference (*P* < 0.0001) between the treated and control group in each study.

Study 7 GM pre-treatment FECs were 748.28 and 783.29 in the control and Credelio Quattro groups, respectively. Following treatment, the FECs on day 10 in the control and Credelio Quattro groups were 478.01 and 1.12, respectively, demonstrating a 99.9% reduction in FECs for the Credelio Quattro group. Similarly, in Study 8, the pre-treatment FECs were 32.82 and 53.42 in the control and Credelio Quattro groups, respectively, and 52.61 and 0.00, 10 days post-treatment, indicating a 100% reduction in FECs after treatment with Credelio Quattro.

In Study 9, nine of 13 dogs naturally infected with *U. stenocephala* demonstrated an adequate infection. The GM worm count in the control group was 7.29 (0–263) with no *U. stenocephala* worms recovered from dogs treated with Credelio Quattro, demonstrating 100% efficacy of Credelio Quattro against adult *U. stenocephala*, with a statistically significant difference (*P* < 0.0001) between the treated group and the control group. Pre-treatment FECs in the control group and Credelio Quattro group were 211.06 and 255.37, respectively. On Day 10 post-treatment, FECs were 111.42 and 0.00 in the control group and treated group, demonstrating a 100% reduction in FEC for the Credelio Quattro-treated group.

## Discussion

Worldwide, dogs of all ages and lifestyles are at risk for hookworm infections. Treatment of puppies and adult dogs is vital to prevent clinical disease and to decrease environmental contamination, reducing the risk of exposure for dogs and humans.

The presented studies demonstrated that Credelio Quattro was ≥ 99.0% effective against L_4_ stages of *A. caninum*, ≥ 99.8% effective against immature adult *A. caninum*, ≥ 99.9% effective against adult *A. caninum*, and ≥ 99.6% effective against adult *U. stenocephala*. Credelio Quattro efficacy results against larval, immature adult, and adult *A. caninum* and adult *U. stenocephala* are comparable to results published for the commercially available oral combination formulation Simparica Trio (sarolaner, moxidectin, and pyrantel) [6]. Additionally, treatment with Credelio Quattro provided a ≥ 99.9% reduction in FEC 10 days post-treatment when administered to dogs with patent *A. caninum* and *U. stenocephala* infections. The action of individual active ingredients did not exhibit any interference when administered together in the combination tablet. Credelio Quattro demonstrated effectiveness against different isolates of *A. caninum* and *U. stenocephala* obtained from geographically distinct regions (USA, Europe, and Africa), underscoring similar susceptibility of these isolates to moxidectin or pyrantel in Credelio Quattro.

Anthelmintic drugs currently approved for treatment of *A. caninum* include febantel, fenbendazole, moxidectin, milbemycin oxime, and pyrantel [3]. Credelio Quattro, a combination tablet, includes both moxidectin and pyrantel (as pamoate salt). Results revealed the individual efficacy of moxidectin was ≥ 90.8%, and pyrantel was 56.9% against the L_4_ stages of *A. caninum*. However, when combined in a single formulation, efficacy of Credelio Quattro increased significantly to 99.8%.

Our results showed that moxidectin was more effective than pyrantel against L_4_ stages of *A. caninum*. This may be attributed to moxidectin’s highly lipophilic nature, which results in prolonged tissue residence where larval stages reside before finally maturing in the intestinal tract. Moxidectin in Credelio Quattro demonstrated a swift absorption rate, with the highest plasma concentration recorded at 15.3 ng/mL within 4.5 h after dosing. In terms of elimination, Beagle dogs exhibited a half-life (T_1/2β_) for moxidectin of roughly 26.4 days [24].

Hookworm infections in dogs can be serious if left untreated, as they can lead to various health conditions. Credelio Quattro is effective in killing hookworms, including L4, immature adult, and adult *A. caninum*, thus terminating the lifecycle of the parasite. As a result, Credelio Quattro not only eliminates gastrointestinal infection in dogs, but also reduces fecal egg shedding in the environment, since adult worms that lay eggs have been eliminated. By effectively eliminating hookworms, Credelio Quattro not only protects dogs from hookworm infections, but also minimizes the risk of environmental contamination, which can serve as a source of zoonotic transmission to humans (CLM) and potential infection to other canine companions. Organizations such as ESCCAP, TroCCAP, and CAPC recommend year-round parasite monitoring and control for dogs [18, 20], with recommendations tailored to lifestyles of dogs in those specific geographies. Veterinarians should encourage their clients to follow these established guidelines whenever possible. Data reveal a notable reduction in the occurrence of common nematode and cestode endoparasites with strategic utilization of broad-spectrum endectocides in pet dog populations receiving veterinary care [25, 26]. Credelio Quattro, a chewable tablet combining four active ingredients, is designed with broad parasiticidal activity against hookworms as well as heartworm, lungworm, roundworms, cestodes, and fleas and ticks to simplify administration and enhance owner compliance through a single convenient monthly oral tablet.

## Conclusions

Credelio Quattro, administered once orally at the minimum dosages of 20 mg/kg of lotilaner, 0.02 mg/kg of moxidectin, 5.0 mg/kg of praziquantel, and 5.0 mg/kg of pyrantel, was safe and effective for the treatment and control of L_4_, immature adult, and adult *A. caninum*, as well as adult *U. stenocephala* in dogs.

## Data Availability

Data supporting the conclusions of this article are included within the article.

## Abbreviations

ANOVA: Analysis of variance
CAPC: Companion Animal Parasite Council
TroCCAP: Tropical Council for Companion Animal Parasites
CLM: Cutaneous larva migrans
EPG: Eggs per gram
ESCCAP: European Scientific Counsel Companion Animal Parasites
FEC: Fecal egg count
L_1_: First-stage larvae:
L_4_: Fourth-stage larvae
GM: Geometric mean
GI: Gastrointestinal
L_3_: Infective third-stage larvae
LS: Least squares
LMPP: Lotilaner, moxidectin, pyrantel, and praziquantel
USA: United States of America
MAR: Morocco
EU: European Union
SA: South Africa
